# Statistical Modelling Investigation of MALDI-MSI-Based Approaches for Document Examination

**DOI:** 10.3390/molecules28135207

**Published:** 2023-07-04

**Authors:** Johan Kjeldbjerg Lassen, Robert Bradshaw, Palle Villesen, Simona Francese

**Affiliations:** 1 Bioinformatics Research Center, Aarhus University, Universitetsbyen 81, 3. Building 1872, DK-8000 Aarhus, Denmark; johan.lassen@birc.au.dk (J.K.L.); palle@birc.au.dk (P.V.); 2 Centre for Mass Spectrometry Imaging, Biomolecular Sciences Research Centre, Sheffield Hallam University, Sheffield S1 1WB, UK; r.bradshaw@shu.ac.uk; 3 Department of Clinical Medicine, Aarhus University, Palle Juul-Jensens Boulevard 82, DK-8200 Aarhus, Denmark

**Keywords:** MALDI, machine learning, documents, ink

## Abstract

Questioned document examination aims to assess if a document of interest has been forged. Spectroscopy-based methods are the gold standard for this type of evaluation. In the past 15 years, Matrix-Assisted Laser Desorption Ionisation–Mass Spectrometry Imaging (MALDI-MSI) has emerged as a powerful analytical tool for the examination of finger marks, blood, and hair. Therefore, this study intended to explore the possibility of expanding the forensic versatility of this technique through its application to questioned documents. Specifically, a combination of MALDI-MSI and chemometric approaches was investigated for the differentiation of seven gel pens, through their ink composition, over 44 days to assess: *(i)* the ability of MALDI MSI to detect and image ink chemical composition and *(ii)* the robustness of the combined approach for the classification of different pens over time. The training data were modelled using elastic net logistic regression to obtain probabilities for each pen class and assess the time effect on the ink. This strategy led the classification model to yield predictions matching the ground truth. This model was validated using signatures generated by different pens (blind to the analyst), yielding a 100% accuracy in machine learning cross-validation. These data indicate that the coupling of MALDI-MSI with machine learning was robust for ink discrimination within the dataset and conditions investigated, which justifies further studies, including that of confounders such as paper brands and environmental factors.

## 1. Introduction

Questioned document examination (QDE) is the area of forensic investigation that aims to determine whether a document has been forged. Types of forgery include, amongst others, identity fraud, banking forgery, and contract forgery, and the modalities of forgery vary too. However, often, authenticity is evaluated by classifying and comparing the paper and/or the ink within the questioned pages of a document [1,2,3,4,5]. In many forensic laboratories, the go-to approach is largely based on spectroscopic methods such as Raman spectroscopy [4,5] and ultraviolet-visible spectroscopy (UV-vis) [6,7,8]. Recently, X-ray photoelectron spectroscopy (XPS) was also proposed [2]. Additionally, several studies have successfully distinguished the ink of specific pen brands by using mass spectrometry (MS) methods including gas chromatography–mass spectrometry (GC-MS) [9,10], desorption electrospray ionisation (DESI) [11,12,13], and Laser Desorption Ionisation (LDI) [14,15,16]. Chemometric methods have proven to be extremely powerful in the handling of data generated to eventually discriminate ink in relation to questioned documents [17,18,19]. However, the vast majority of studies have been qualitative in nature; for example, PCA clustering and spectral differences have been used to visually discriminate ink profiles. As such, quantification of certainty and assessment of the influence of confounders, such as the time that has passed since writing [1,20,21], remain areas of forensic analytical interest and development. One notable study was undertaken in a round-robin fashion within EU COST Action CA16101. There, a variety of physical, spectroscopic, and MS methods were employed by 17 laboratories across Europe to *(i)* address questions in relation to document forgery [22] and *(ii)* compare the information value of each technique from both a qualitative and a quantitative perspective. In that study, some of the questions specifically asked to differentiate and classify ink were designed to assess in which way and where forgery occurred. Whilst these questions have been answered with varying degrees of success, no laboratory has yet been able to address the question of ink age after the time of deposition.

This study has shown that most analytical methods fluctuate in classification performance between labs and highlighted the importance of statistical modelling to rationalise data, discover trends, and ultimately differentiate ink in both a qualitative and a quantitative way. Although several other studies have statically assessed the performance of pen classification by using machine learning or distance-based statistics [13,16,23], further efforts should improve the reliability of chemometrics in ink discrimination in order to generate a real operational impact.

**Figure 1:**
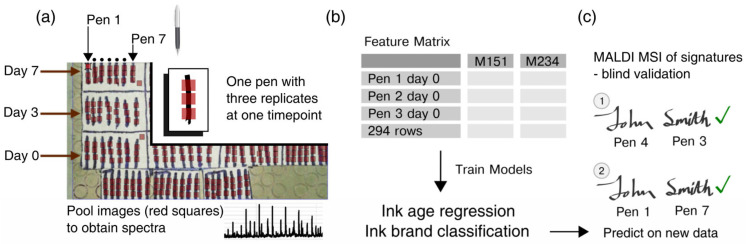
Study workflow. (**a**) MALDI-MSI data acquisition of imaging areas (red squares) on ink lines: each line represents a different pen’s stroke, and each box/area represents a given sampling day (day 0 to day 44 over a total of 14 time points). Red squares represent imaging areas of 1 × 1 mm, collected as triplicates for each pen at each time point. Areas not occupied by any ink were used as blanks. (**b**) Model training: mass spectra extracted from the images in (**a**) were used to generate a feature (peak) matrix using MALDIquant, which was then used for regression and classification models. (**c**) Validation: the classification model was validated using a MALDI-MSI dataset from signatures simulating forgery on a new sheet of paper (same brand).

In this study, a combination of Matrix-Assisted Laser Desorption Ionisation–Mass Spectrometry Imaging (MALDI-MSI) and chemometrics was used to investigate the hypothesis that this combined method could differentiate the ink of seven ballpoint pens. MALDI-MSI was selected due to its demonstrated ability to provide intelligence in an operational context in regards to finger marks [24,25,26] and blood [27,28,29] as well as hair [30]. In its profiling modality, it has also shown promise for ink analysis [31,32,33]. This study therefore intended to explore the possibility of extending the use of MALDI-MSI to another type of evidence in order to promote this technology as a multievidence analytical platform in a forensic and operational environment. Moreover, the combination with machine learning was designed to obtain both a qualitative and a quantitative assessment of the hypothesis and increase the robustness of the answer. For this study, we developed a stepwise analytical workflow (Figure 1) whereby (Figure 1a) averaged mass spectra from the ink of seven black gel pens were exported from MALDI-MSI data to assess the possibility of differentiation between gel pens as well as evaluation of the effect of time since ink deposition (“age”) on pen classification; (Figure 1b) regression models were implemented to evaluate the age effect to then progress to classification models of the pen brands; and (Figure 1c) the classification model was applied on a blind MALDI-MSI dataset consisting of five signatures made with different pens in order to validate the methodology, assess the robustness of the statistics, and demonstrate the feasibility of the overall method for ink classification.

The results showed that coupling MALDI-MSI with machine learning is robust for ink discrimination, with a 100% correct identification rate achieved for the validation signature dataset based on the same pens and aging conditions as the training set. It was also concluded that age (time since ink deposition) does not impact pen classification. However, neither of the above conclusions can be extended beyond the dataset investigated or the aging time and the environmental conditions selected for this study. Hence, this study supports further investigations of factors, including, in a non-exhaustive list, paper brands, light exposure, and temperature, as well as a wider range of new pen brands and pens from different production batches.

This study also briefly investigated the possibility of dating ink deposited at different times. However, the dating issue remains elusive for ink, as it is for other types of evidence, such as finger marks, blood, and other biological traces.

## 2. Results and Discussion

With the overall objective of developing a MALDI-MSI–machine learning combined method for ink and gel pen classification, the initial aim was to *(i)* firstly verify that it was indeed possible to discriminate between gel pens and *(ii)* secondly assess the method’s robustness by challenging it with ink aging. For this initial feasibility study, a limited number, rather than a comprehensive library, of pens was selected. In particular, seven black gel pens were selected, the ink of each of which was indistinguishable by the eye (except for pen 6 after MALDI matrix deposition, which bled into the paper and changed colour; data not shown). The MALDI-MSI data of these gel pen ink strokes were acquired in triplicate from 10 × 10 pixels areas per stroke across 14 time points (0, 3, 7, 9, 13, 17, 20, 24, 28, 31, 35, 38, 41, and 44 days) spanning 44 days (mean = 22.1; SD = 14.2) (Figure 1). From each replicate image, an average mass spectrum was exported, yielding 294 observations in total. The mass spectra were preprocessed with MALDIquant (see Methods Section 3.3.1) and yielded 3739 features after the exclusion of peaks with high variances in blanks compared to the ink samples [34]. Finally, the feature matrix was preprocessed for statistical analysis using robust row normalisation (see Methods Section 3.3.1). Already, in visual inspection, the MALDI-MS spectra appeared very different, with unique peaks for the ink of each pen (Figure S1, Supplementary Materials). Most spectra contained polymers—a common part of the aqueous medium component in gel pens [35]—which heavily populated the mass spectra of especially pen 5’s (Pentel Energel BL77-A) ink across the entire *m/z* acquisition range.

**Figure 2:**
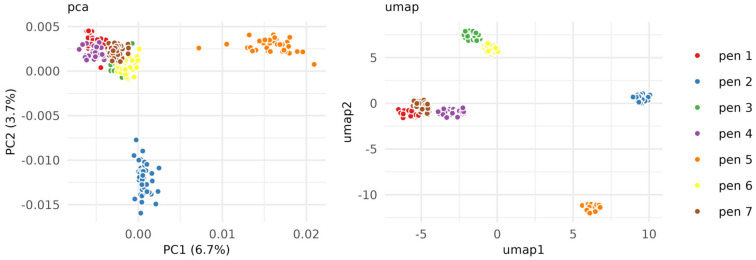
Unsupervised modelling of ink data. PCA and UMAP show clear separations between pen types.

Unsupervised data analysis confirmed spectral differences by clustering each pen type together across all time points. Both PCA and UMAP were used on all 297 samples and 3739 features. The PCA separated pen 2 (Pilot G1-100) and 5 (Pentel Energel BL77-A) from the other pens, whereas the UMAP (another unsupervised clustering method suited to non-linear data) clustered all pens due to its nearest neighbour based properties (Figure 2). PCA measures the dimensions explaining most variance which might be defined by the highest intensity peaks across all samples (and noise), whereas UMAP is suited for binary peaks (existing vs. not existing) that constitute the discrete differences between pens. No aging gradient pattern was observed in either the UMAP or the PCA of the full dataset (Figure S2), suggesting that age does not impact pen differentiation.

### 2.1. Determination of Ink Age (Time since Ink Deposition)

As a natural follow up to the study challenging the model with ink age, an investigation of the possibility to date ink (time since deposition) using machine learning was undertaken. For this purpose, cross-validated elastic net regression was used [36], as it reduces the number of used features (by removing covarying features) and provides a small set of high-quality predictors.To avoid data leakage, the hold-out partitions were complete triplicates from a single time point, predicted by a model trained on the remaining time points. Each pen’s ink was modelled individually, as the unsupervised learning showed no evidence of common age markers. The single-pen age regression models yielded poor performances, with no or weak associations with age, suggesting that this predictive task was not achievable for the dataset investigated (Table 1).

**Figure 3:**
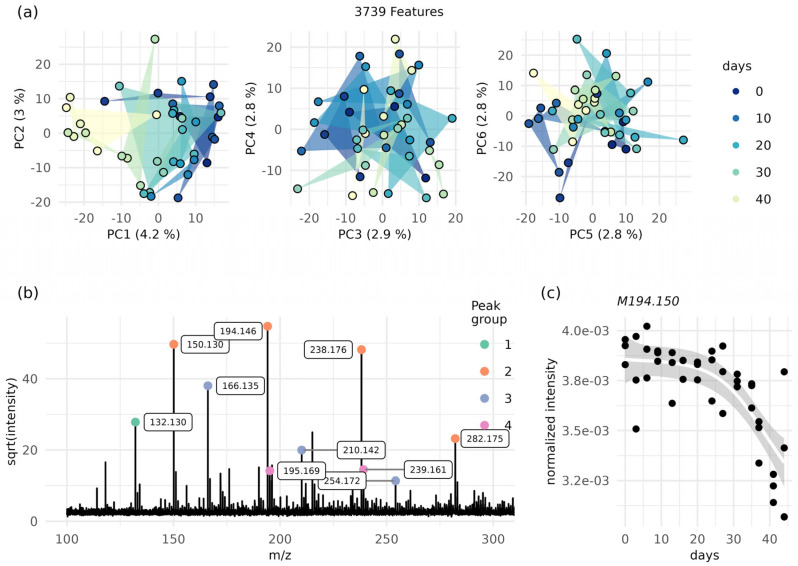
PCA- and FDR-corrected Spearman tests show aging PEGs in pen 1. (**a**) PCA of the pen 1 profiles over the period of 44 days; the first six principal components are shown. Triplicates are connected by triangles. (**b**) Univariate analysis highlights the aging of PEGs; FDR values from Spearman correlation analysis display a hotspot of correlated peaks, originating from a PEG, presenting itself as multisignal Gaussian distribution distanced by 44.05 mass units. (**c**) The highest intensity peak from the correlated PEG (*m/z* 194.146) shows non-linear aging (line: generalised additive model).

Finally, age regression was performed on all pens simultaneously to assess if the increased sample size benefitted the power. However, this model performed worse than random model (i.e., the standard deviation when using mean age as the baseline model) (Figure S3). Subsequently, an investigation of whether the average MALDI-MS spectra of the ink contained any “aging ion signal” (or age markers) using the Spearman correlation test followed by false discovery rate (FDR) correction of the *p*-values (Table 1) was undertaken. Pen 1 (ASDA gel pen) and pen 4 (Uniball Signo UMN 207) had more than 100 correlated features at false discovery rates (FDRs) of less than 0.01, whilst pen 7 (WHSmith rollerball gel pen) only had 44 significant peaks. The varying numbers of correlated features suggest that each ink brand contains a unique set of aging features, which further highlights the challenge of making a global aging model of all pens used in this study. Despite poor machine learning performance, the FDR values indicated some degree of association between the features (peaks) and age; on this basis, PCA was performed on pen 1 (ASDA gel pen) to investigate the unsupervised data structures (Figure 3). The first principal component (PC1) revealed an aging pattern with tight clustering of young samples and large distances between older samples (Figure 3a), suggesting that aging contributed to most of the variance in pen 1 (ASDA gel pen). Despite the clear aging gradient across the PC1 axis, only 4.2% of the total variance was captured by PC1, indicating that a large proportion of the data showed no clear/defined pattern. Finally, the PCA of the remaining pens revealed that pens 4 (Uniball Signo UMN 207) (PC1) and 5 (Pentel Energel BL77-A) (PC3) also contained aging gradients, whilst the remaining pens showed no or weak patterns (Figure S4).

Finally, it was observed that ten of the top 20 correlated features in pen 1 (ASDA gel pen) belonged to a polyethylene glycol (PEG) and its adducts (Figure 3b), all of which havd FDRs ranging from $1.7\times 10^{-5}$ to $5.7\times 10^{-8}$. The PEG ion signals showed a negative correlation to age, indicating time dependent degradation. This observation agrees with Sun et al. [35], although in our study, the decreased PEG-related ion intensity occurred in the last 10–20 days of the experiment. The degradation of the PEG was non-linear, having the biggest decline after 20 days and matching the pattern shown by the PCA. No PEG-related peaks underwent ion intensity increases. To ensure that the ion signal decrease did not originate from a general decrease in the total ion current (TIC) values over time, we correlated the TIC for each replicate at each time point with the corresponding age and found no trend (Figure S5). Based on the evidence from the ML performance, the FDR values, and the single-pen PCA investigations, it is possible to conclude that aging does not greatly affect the ability to correctly classify ink and discriminate pens using MALDI MSI as the analytical technique. However, this conclusion is only valid for the given period and storing conditions investigated. Indeed, environmental factors most likely also influence molecular degradation, and, as such, longer aging times and a range of environmental factors, including the effects of light and darkness, temperature, humidity, and storage conditions, should be investigated. However, the assessment of these variables’ effects (often cross-talking) is complex and requires both comprehensive sampling and appropriate sample sizes. To the best of the authors’ knowledge, only one study has validated its aging models with new data, but it obtained reasonable predictions in only two out of five validation pens, thus highlighting that aging (degradation) is compound- and therefore pen-dependent [37]. The data obtained and described in the present study support this conclusion, as it was found that the number of aging features was highly variable between pens. Whilst absolute ink dating remains one of the Holy Grails of forensic science (as do blood and finger mark dating), relative age may be also interesting and more robustly assessed, as this concept refers to the question of whether one ink stroke is older than another [35]. The relative age might cancel out the confounding factors [38] because questioned signatures often are stored under the same conditions (together), thus making it possible to obtain a measure of the two signatures being of the same age—e.g., if high-temperature storage accelerates ink aging, it might affect a forged and an original signature equally, which would counteract the effect of age.

### 2.2. Ink Classification

**Figure 4:**
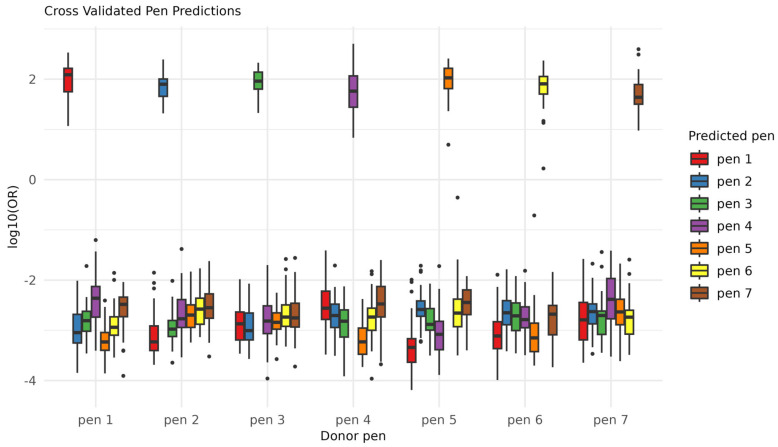
Machine learning discrimination of ink. Log odds ratios of the predictions returned by the model. Each observed category (*x*-axis) is predicted correctly and with high certainty (separation in *y*-axis).

An elastic net logistic regression was performed using the “leave one triplicate out cross-validation” for performance estimation (Figure 4).

As the data contained one class per pen (the pen ID), the classification was multinomial and returned seven probabilities for each prediction: one for each pen ID. These probabilities reflected the likelihood of each class, and the sum of all of the probabilities was 1.

The cross-validated performance yielded 100% accuracy. As there were seven classes, the probability of randomly guessing one class correct was 1/7 (~14%), which emphasizes a superb ML performance. Additionally, the class probabilities were highly separated between the “true” and “false” classes, indicating either extremely reliable or overfitted predictions. The predictions were converted to log10 odds ratios, showing that most of the classifications were predicted at a log odds ratio of 2; that is, the model predicted the correct class with a probability of ~100 ($10^{2}$) times higher than the other possibilities (corresponding to ~99% vs. ~1%). The technical variabilities of the features included in the classification model (*n* = 102) were assessed using the distributions of pairwise correlations of the technical replicates vs. the pairwise correlation of the non-replicates, as well as the differences in relative standard deviation (RSD) between the technical replicates and the non-replicates; overall, the important features were stable (Figure S6).

### 2.3. Method Validation

**Figure 5:**
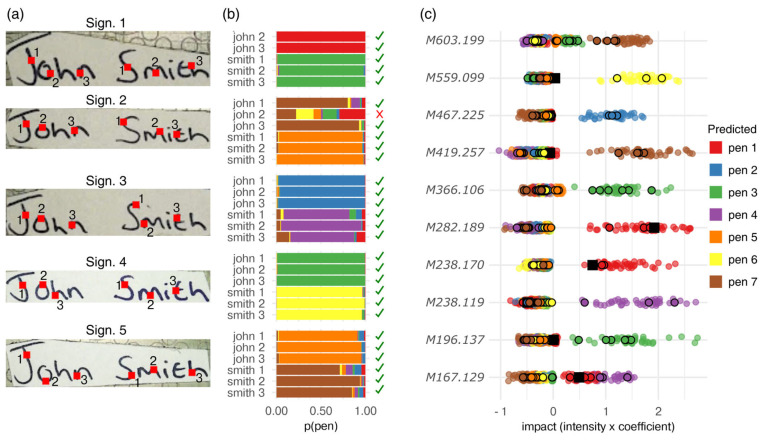
MALDI-MSI-ML combined approach on signatures generated by different pens (blind validation study). (**a**) Signature sets where the first and last names were (potentially) generated by different pens, also showing the locations of the triplicates collected from each MALDI-MS image (full signature imaged) (red squares for illustrative purposes, position approximate). (**b**) Predicted classes of each replicate and correctness of classification. The *x*-axis represents the predicted probabilities with a sum of 1; p (pen). (**c**) Cross-validated impact of each compound on the pen classification. One point represents one sample. Black-bordered circles are the blind validation data, and the black square is the single incorrect prediction (signature 2, John 2).

A validation study of signatures that simulated different scenarios of forgery was conducted in a blind fashion to *(i)* strengthen the results showing the feasibility of MALDI-MSI for ink/pen discrimination, *(ii)* ensure experimental reproducibility, and *(iii)* demonstrate that there was no overfitting of the training data (Figure 5).

Blind to the analyst, a volunteer generated a set of five signatures using different pens for the first name and surname of each signature on a new sheet of paper (Figure 5a). The volunteer was also given the choice to use the same pen for the entire signature. Once deposited, the signatures were stored for 10–14 days (see Methods) before being analysed with MALDI-MSI. Regions of interest of 10 × 10 pixels of the signatures were exported and averaged to obtain measurements matching the format of the training data (Figure 5a). As with the training data, these regions of interest may have covered small areas with no ink deposited, but because of the homogeneity in the data (training vs. validation), the model accounted for this variability. Both the first names and surnames were measured in three different areas which then were predicted by the trained classification model (Figure 5b). As observed with the previous dataset, the classification model returned seven probabilities (one per pen ID) for each area. In most cases, the each classification had a single, very certain class, such as signature 1, where “John” was assigned to pen 1 (ASDA gel pen) and “Smith” to pen 3 (Tesco gel pen). Only pen 7 (WHSmith rollerball gel pen) yielded uncertain probabilities for the predictions in signature 2 (John) (max replicate log odds = 1.1) and signature 5 (Smith) (max replicate log odds = 1.2)—i.e., the most certain replicate was only 10 times more likely to be pen 7 (WHSmith rollerball gel pen) than any other pen. The validation data only yielded one disagreeing prediction, namely a single measurement of “John” in signature 2, and despite this, the two other replicate measurements agreed.

In two previous studies by Weyermann et al. [15,16], distance-based statistical methods instead of machine learning were used to distinguish pen types. This method measured the Euclidian (or correlation-based) distance between two pens (using LDI spectra) and generated two distributions of identical pairs vs. non-identical pairs. This approach is likely to be more transferable to new observations of unknown pen brands, as it does not rely directly on training data; one could generate the similarity distribution from two signatures and assess whether the distribution is bimodal. However, this distance method was not evaluated on new (or blind) data, in contrast to the method that is being proposed in the present study. Finally, our model’s reliability was assessed by investigating the feature-wise impacts on the predictions (Figure 5c). The feature-wise impacts were calculated by multiplying the model coefficients with the feature intensities, yielding a metric that would increase proportionally to the modelled probability of a given observation. Specifically, the feature-wise impacts allowed direct comparison of the training data and the validation data to ensure that the features and their influence on the model were not susceptible to the batch effect or similar biases. As the feature-wise impacts from the training data (cross-validated) and the blind validation data were behaving identically, high reliability and reproducibility could be assumed—although the single false prediction (black square) mistakenly had high impact features corresponding to pen 1 (red distributions). This observation highlights the importance of using multiple predictors to obtain reliable log odds ratios, as the predictors that negatively impacted the log odds ratio (prediction certainty) were not located in the top 10. To sum up, the log odds ratio and feature impacts can be used to evaluate the reliability of predictions. Hence, if the model were to predict new ink brands that have not yet been included in the data, the log odds ratios and feature impacts may be uncertain or inconsistent. Using the majority scoring system, “John” in signature 2 was associated with the use of pen 7 (WHSmith rollerball gel pen) whilst the other predictions had consensus between the three measurements. Upon this final evaluation of the predictions, the true classes were revealed, and all signatures were correctly classified. However, in any real-world application, it may be wiser to refrain from concluding on a signature when having disagreeing predictions and the generation of additionally pooled MALDI spectra from the image would be desirable to provide further evidence. Whilst this study cannot be immediately generalisable to other pen brands, given the predictive power shown in the validation study, it is possible to speculate that these results may likely generalise well. However, before the investing of resources in expanding the number of modelled pen brands, confounders such as paper brands and environmental effects must be assessed to ensure that no biases will affect the ML performance. Regularised linear models were employed, as these provide predictors of maximum relevance and minimum redundancy as well as high inference and extremely accurate predictions. Ensemble models such as random forest or gradient boosting would only decrease the model insight, though they might be better for modelling the non-linear trends of age. 

The use of MALDI-MSI to detect document forgery via ink analysis was published for the first time by Fischer et al. [22]. Whilst in that study, this technique did detect where the forgery occurred, the assessment was manual and purely qualitative. This paper shows that the combination with ML strengthens the capability of this technique in the application to ink analysis. Whilst, undoubtedly, ambient mass spectrometry methods, such as DESI-MSI, are less destructive and could be applied in a much faster way with fewer technical constraints, this paper shows that investing in a technology such as MALDI-MSI is worthwhile from the point of view that it provides the user with the opportunity to investigate several types of forensic evidence, three of which (fingerprints, blood and hair) are already associated with high technology readiness levels.

## 3. Materials and Methods

### 3.1. Materials

An α-Cyano-4-hydroxycinnamic acid matrix, red phosphorus, and trifluoroacetic acid were purchased from Sigma-Aldrich (Poole, UK). Acetonitrile (ACN) was purchased from Fisher Scientific (Loughborough, UK). Double-sided conductive carbon tape was obtained from TAAB (Aldermaston, UK). Seven black gel pens from different brands were used, including a Uni Ball Signo, a UMN-207, a Pentel Energel BL77-A, a Pilot G-1, a Tesco home office gel pen, a Zebra Sarasa, a WHSmith rollerball gel pen, and an ASDA gel pen. Woodland Trust (75 g, uncoated) paper was used for the deposition of ink.

### 3.2. Methods

#### 3.2.1. Generation of Sample Datasets 

The ink sample dataset was generated by drawing one line each at 2–4-day intervals over the sampling period of 44 days (0, 3, 7, 9, 13, 17, 20, 24, 28, 31, 35, 38, 41, and 44 days) on a sheet of paper (Table 2). Before ink deposition, each pen was primed to ensure consistent ink deposition. The writing force was not controlled to simulate normal (or varying) writing. After the sampling period (aging time), the samples were analysed with MALDI-MSI in one go, as described in Figure 1 and Section 3.2.1.

The signature samples were collected by asking an individual to generate 5 signatures on a new sheet of paper (not the same piece as the training data). For each signature, the individual was given the choice to either use different pens for the first name and the surname or use the same pen throughout the whole signature. The signatures were aged for 10 (signatures 4 and 5) and 14 days (signatures 1, 2, and 3), respectively.

#### 3.2.2. MALDI Matrix Deposition

Upon method optimisation consisting of testing different combinations of temperatures, flow rates, layers, and α-Cyano-4-hydroxycinnamic acid (α—CHCA) matrix concentrations (Table S1), the aged ink strokes (Figure 1a) were excised and stuck onto the MALDI target plate using double-sided conductive carbon tape. Ten mg/mL of the α—CHCA matrix in 70:30 ACN: 0.5% TFA was sprayed onto the paper using an M3+ Sprayer (HTX technologies, LCC, Chapel Hill, North Carolina, US), with the nozzle temperature set at 75 °C, the flow rate set at 100 µL/min, and 4 layers deposited at a pressure of 10 psi.

#### 3.2.3. MALDI-MSI of Ink from Black Gel Pen Strokes and Signatures

The MALDI-MSI data were acquired with a Synapt G2 HDMS MALDI qTOF system. For each stroke, three 1 mm^2^ images were acquired at a spatial resolution of 100 µm. The instrument was set to positive ionisation mode in the *m/z* range of 100–1500 Th and calibrated using red phosphorus over the same range. The laser power was set at 280 a.u. and fired at a repetition rate of 1 kHz and an accumulation time of >1 s, resulting in 1000 scans per pixel. Finally, five blanks containing only matrix-coated paper were acquired from the surface. The MALDI-MSI of the signatures was acquired using the same settings as the training data, except for varying spatial resolutions of 100 (signature 4), 120 (signatures 1, 2, and 3), and 150 (signature 5) µm, respectively, because of instrumental time constraints given by the size of the signatures. 

### 3.3. Statistical Analysis and Software 

#### 3.3.1. Data Preprocessing

The raw MSI training data were converted with Proteowizard to mzML files whereas the raw MSI validation data were converted to the imzML format within the Waters HDI software (imzML files retain image metadata used to point out regions of interest) [39]. The imzML and mzML spectra were then preprocessed in the R packages MALDIquant and MALDIquantforeign [34]. Firstly, MALDI-MS spectra were extracted from the regions of interest (10 × 10 pixels) within the MALDI-MS validation images; subsequently, all regions of interest (including the training data) were averaged (pooled), log transformed, smoothed, baseline corrected, aligned, and given peak detection to obtain the feature matrix. For peaks to be included in the dataset, they had to have a minimum occurrence of 1% (equivalent to one triplicate) and a signal to noise ratio (SNR) of 3. All other MALDIquant parameters can be found in the data processing notebook at github.com/JohanLassen/ink. This type of workflow means that the preprocessing steps must be re-run every time new samples are added to the dataset. Before the statistical analysis, the data were also normalised as the final step in the preprocessing. Here, robust row normalisation was used to make sure all samples belonged to the same distribution (i.e., independent of the TIC and similar technical biases). This method is very similar to TIC normalisation, but only includes stable feature intensities, as has been described and applied in previous studies [40,41]. Finally, features were omitted if their median absolute deviations (MADs) were less than 1.5 times higher than the MAD of the blanks.

#### 3.3.2. Statistical Machine Learning

The statistical analysis was performed using base R functions and the R packages glmnet and umap [36,42]. The glmnet package was used for the elastic net modelling [36], which is a penalised linear regression. Penalisation means that every time a linear model includes/increases the weight of a feature coefficient, the model is penalised. The penalisation increases the model’s loss function, which is subject to minimisation during model fitting. Hence, a feature’s information contribution must have reduced the loss function more than the penalisation increased it. Consequently, features with high collinearity were excluded, as they provided no gained information to the model. Also, the high dimensional setting (#samples << #predictors) causes traditional linear models to overfit, whereas the elastic net is forced to reduce the number of predictors and will (most likely) not overfit as much. Finally, the fitted elastic net model is as interpretable as the linear model.

The elastic net models were fitted using “leave one triplicate out cross-validation” with all samples from an entire time point as hold-out samples [37]. For each fold of the cross-validation, the blind validation data were predicted, and after the cross-validation process, the predictions of each blind validation observation were averaged into one final prediction (per measurement area). Feature-wise impact distributions were calculated during the cross-validation by multiplying the trained model coefficients with *(i)* the cross-validation hold-out feature intensities’ highest coefficients to obtain the training distribution and *(ii)* the blinded validation feature intensities to obtain the validation distribution (Figure 5c). For the analysis of the impact of age (time since ink deposition), glmnet was also used in the machine learning regression and Spearman correlation was used in the univariate analysis of the compounds from all pens. The FDR-corrected values were calculated based on 3739 tests (one test per feature) for each pen.

The technical variability was assessed by calculating all pairwise Pearson correlations of technical replicates and non-replicates using only the important features from the classification model (*n* = 102). Also, RSDs were calculated for the technical replicates (same time and pen) and non-replicates (same time, but different pens) to assess their feature-wise technical variability.

## 4. Conclusions

This study shows that MALDI-MSI can be a viable or at least complementary alternative technique to generate useful data from ink in the context of questioned documents. Especially when combined with machine learning, this technique has proven to be robust against ink age as a challenging factor in the short span of approximately 44 days and within the constraints of the only one set of environmental conditions employed. By always collecting 10 × 10 pixels of imaging data and keeping all other instrumental conditions constant, the imaging modality ensures high homogeneity between the training and validation data. Furthermore, the training data were analysed in one sitting, thus minimising instrumental variability and variability originating from different sample sets prepared at different times. However, the blind validation study demonstrated model robustness between sample sets. Whilst robust, MALDI-MSI is semi-destructive, as for it, a document would need to be altered by application of the matrix, excision, and consequent micrometric superficial removal of molecules for analysis. Alternative ambient mass-spectrometry-based techniques, such as DESI, are less destructive, as DESI does not require a matrix coating but may still require excision of the relevant document area. Notwithstanding, given the results obtained, the methodology developed, which was validated in a small-scale blind study, warrants further studies in order to develop a robust MALDI-MSI-based methodological framework for questioned document examination based on ink analysis.

## Figures and Tables

**Table 1:** Age-correlated features with false discovery rate < 0.01 and root mean squared error of the regression model.

Brand	Pen ID	No. of Features with FDRs < 0.01	RMSE (Days)
ASDA Gel Pen	1	122	13.3
Uniball Signo UMN 207	4	117	12.8
Pentel Energel BL77-A	5	68	14.4
Pilot G1-100	2	66	12.9
Tesco Gel Pen	3	64	14.9
Zebra Sarasa	6	51	14.9
WHSmith Rollerball Gel Pen	7	44	15.1

**Table 2:** Sheets, samples, pens, and time points used per dataset.

	Training Data	Validation Data
# of Paper Sheets	1	1
# of MALDI-MSI Samples	294	5
# of Pens	7	Up to 7 (blinded)

The # symbol stands for “Number”.

## Data Availability

The code, raw data, and accompanying documentation are available at https://github.com/JohanLassen/ink (accessed on 4 May 2023) and can be downloaded, together with dependencies, to R as a package using remotes::install_github(“JohanLassen/ink”).

## Supplementary Materials

The following supporting information can be downloaded at: https://www.mdpi.com/article/10.3390/molecules28135207/s1, Figure S1: Pooled spectra of each pen; Figure S2: Unsupervised learning coloured by age (days); Figure S3: Cross validated results from age regression on all pens; Figure S4: PCA of all pens coloured by age; Figure S5: Pen 1 TIC correlation to age (days); Figure S6: Technical replicates indicate that the technical variability is notably lower than the signal-variability of the features used in the classification model; Table S1: HTX matrix configurations.
