# Supplementary material for: Statistical Modelling Investigation of MALDI-MSI-Based Approaches for Document Examination
Source: Molecules. 2023 Jul 4;28(13):5207. doi: 10.3390/molecules28135207 (PMC10343405; doi:10.3390/molecules28135207)
Supplement: Supplementary file 1 [file molecules-28-05207-s001.zip › molecules-2384568-supplementary.pdf]

Supplementary Material

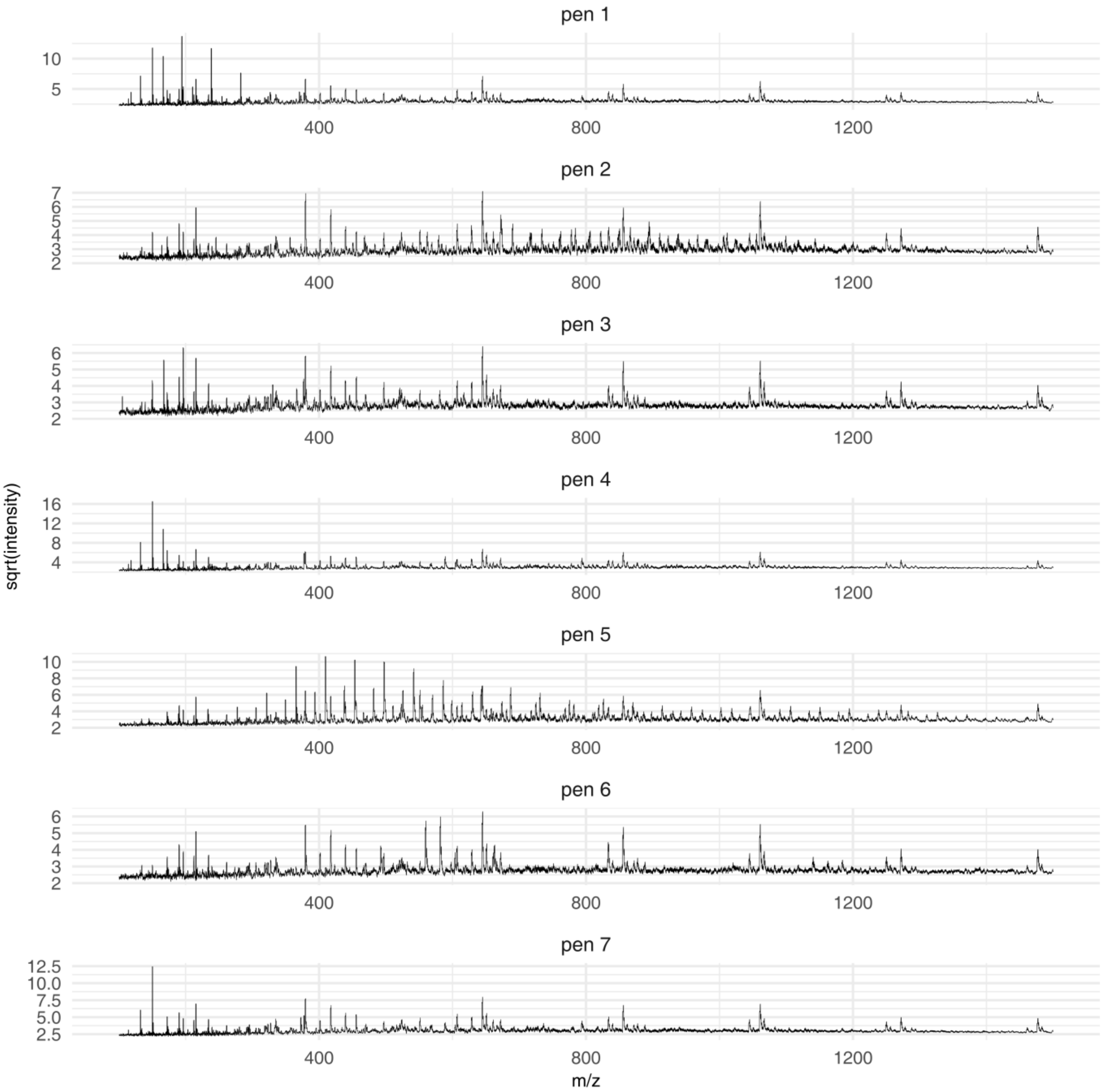

Figure S1. Pooled spectra of each pen. All triplicates across all days were pooled to one averaged spectrum per pen.

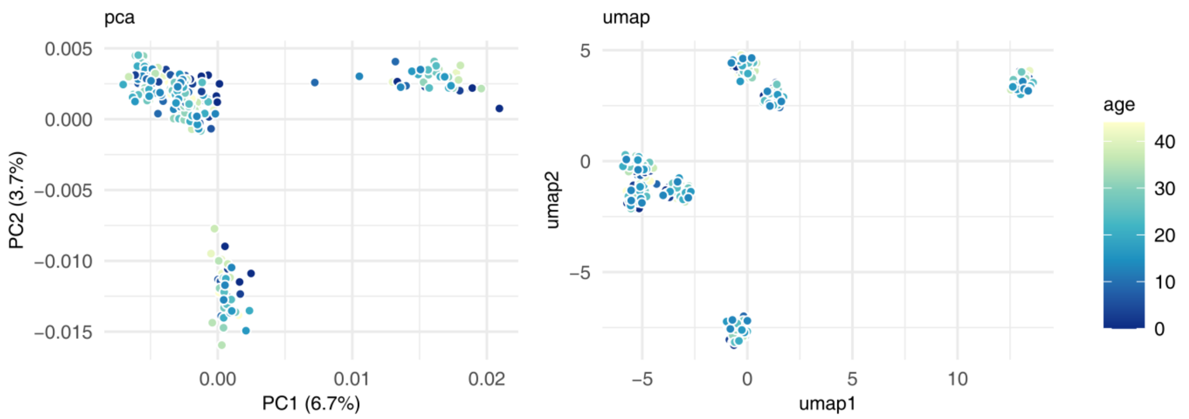

Figure S2. Unsupervised learning coloured by age (days). Same values as Figure 2 (main text) but coloured with age. No aging gradients are observed.

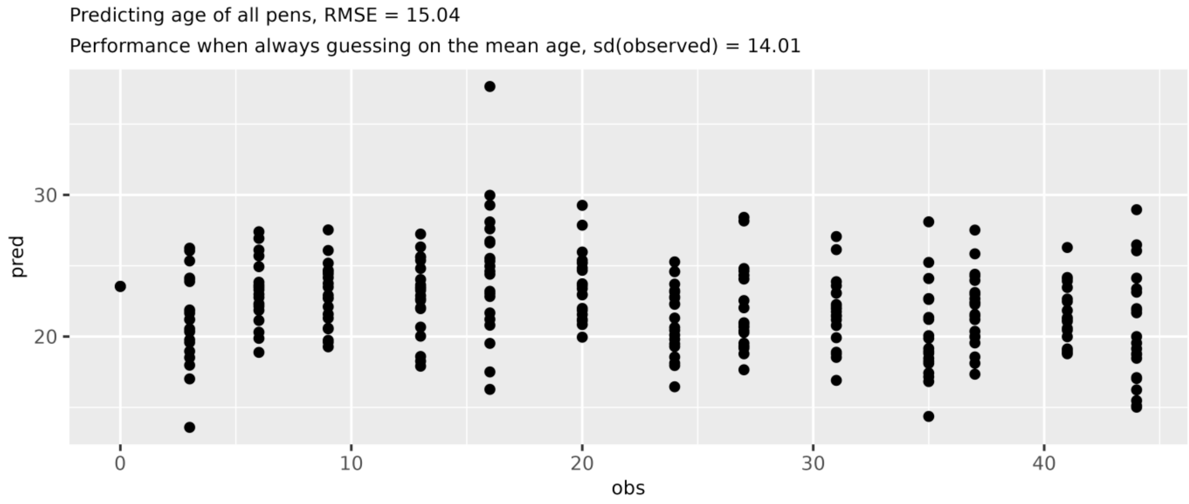

Figure S3. Cross validated results from age regression on all pens. All pens modelled together using elastic net regression.

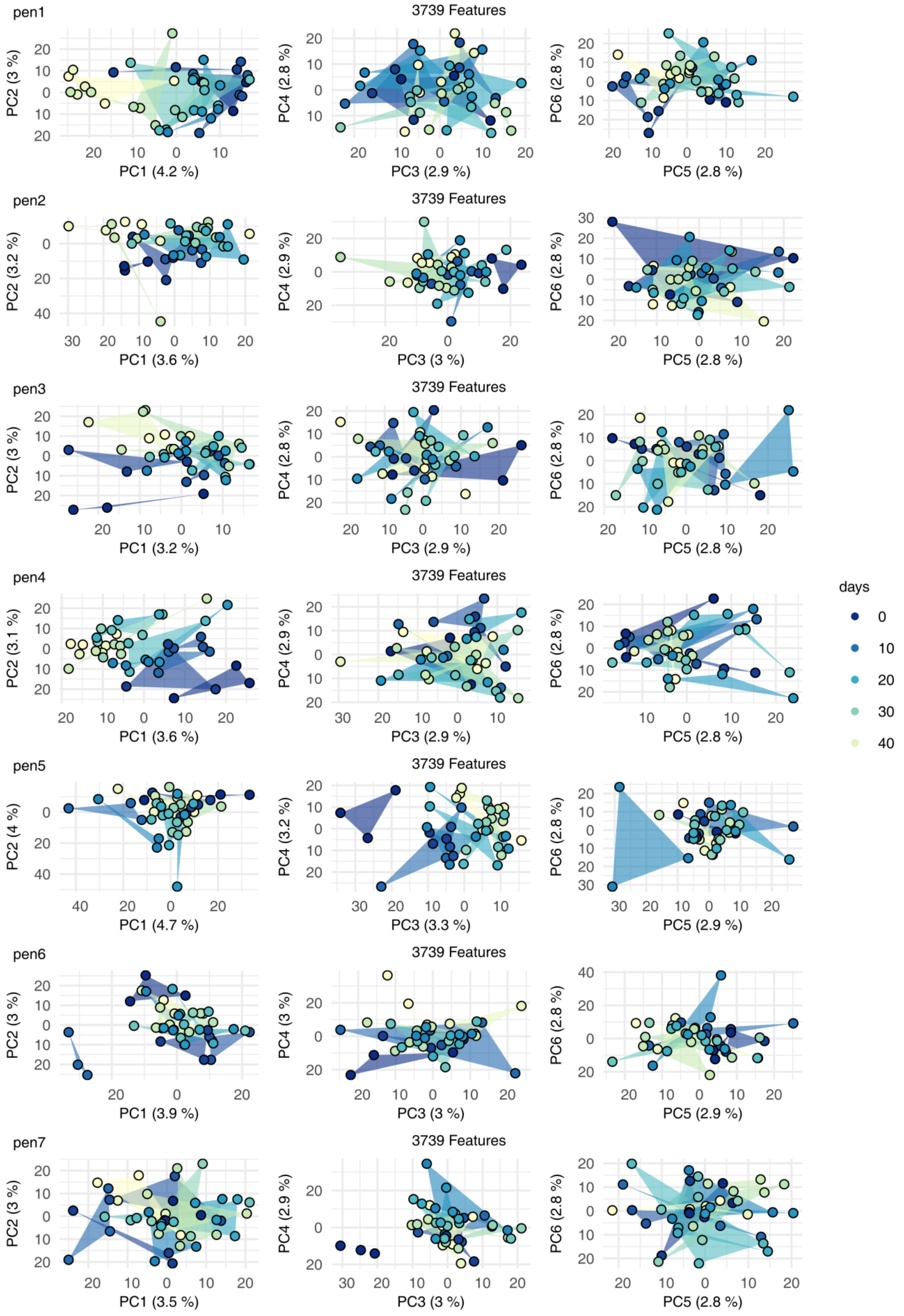

Figure S4. PCA of all pens coloured by age. Each pen modelled separately in PCA, followed by visualization of the first 6 components and coloured by age. Shades/triangles represents technical replicates.

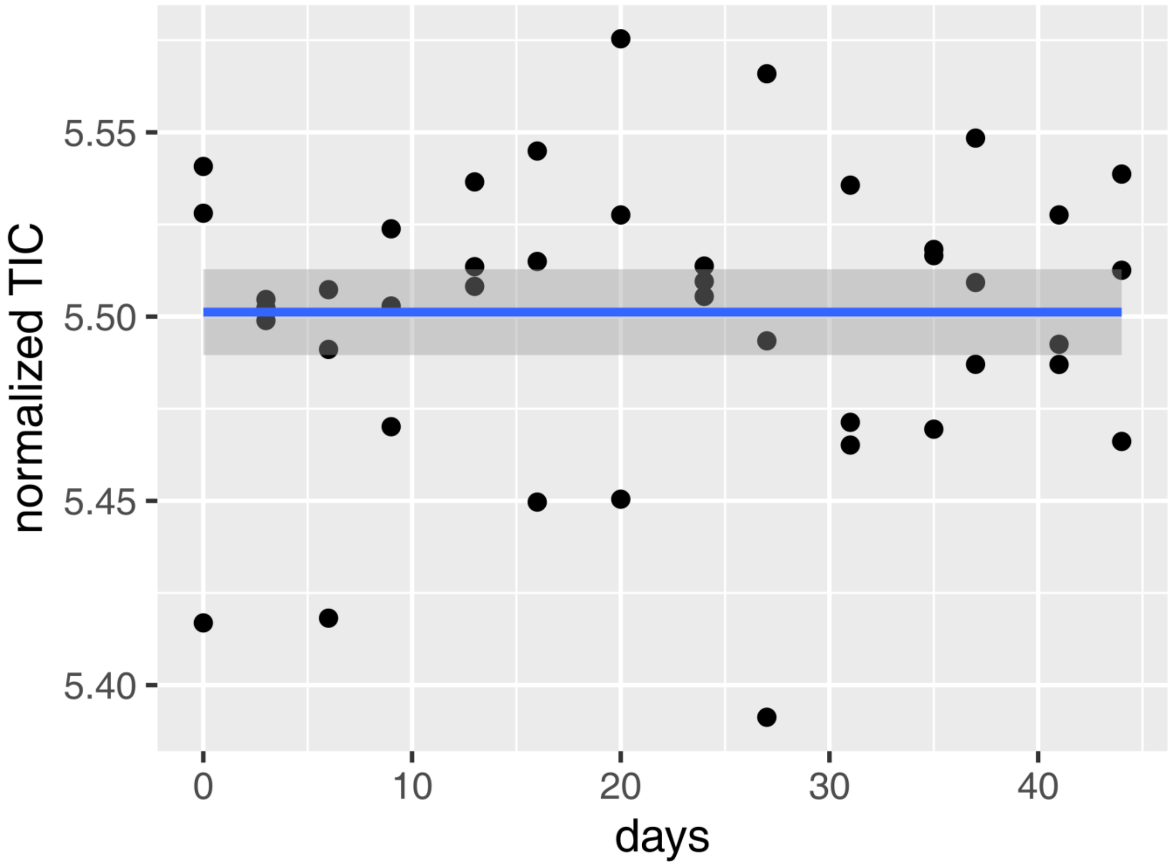

Figure S5. Pen 1 TIC correlation to age (days). No correlation suggests that the overall spectra does not decrease over time.

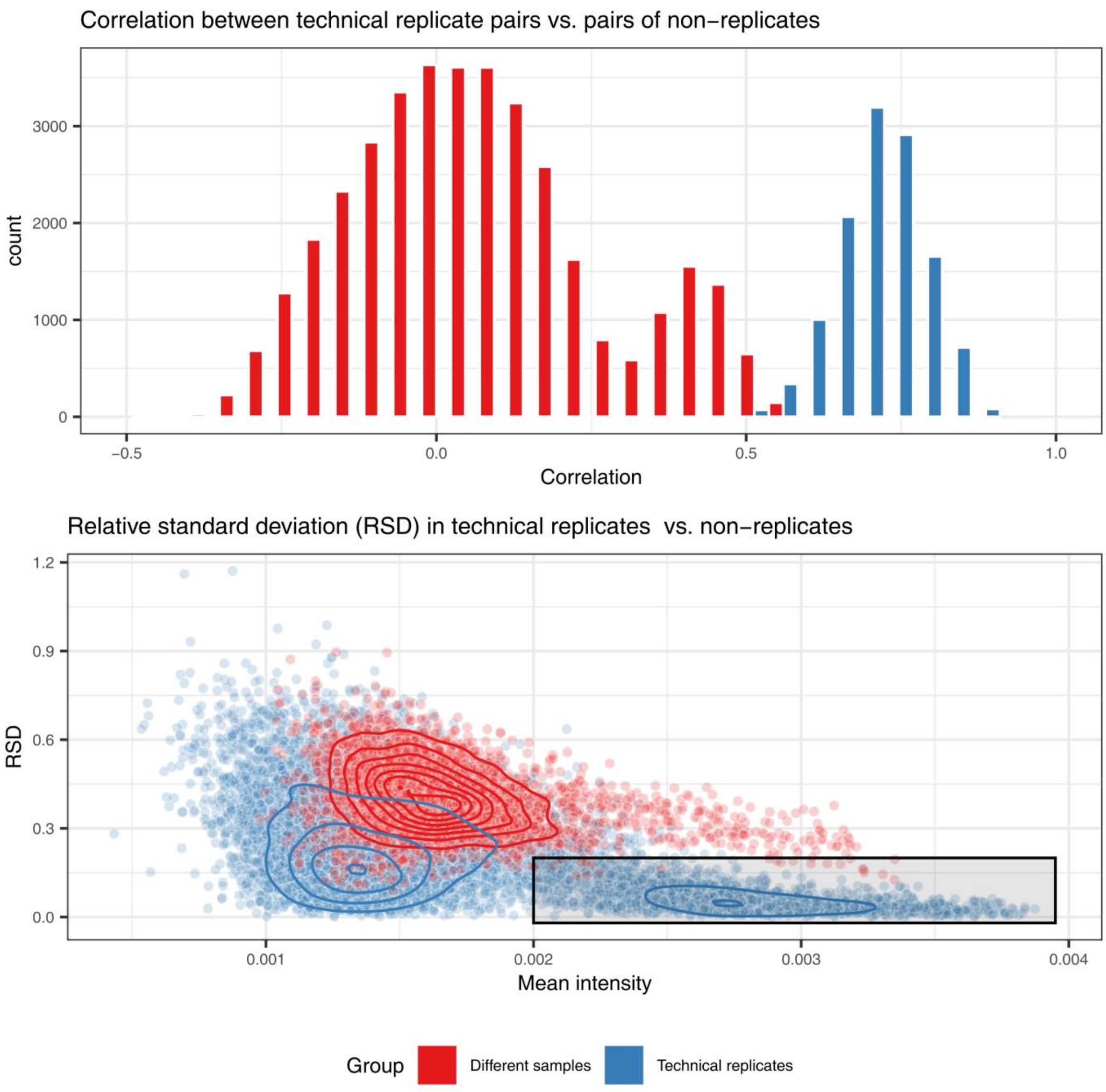

Figure S6. Technical replicates indicate that the technical variability is notably lower than the signal-variability of the features used in the classification model. Top panel: pairwise correlations of technical replicates (same pen and time) are higher and almost completely separated from the pairwise correlations of non-replicates (different pens). Bottom panel: relative standard deviation (RSD = SD / mean) of technical replicates (same pen and time) versus the RSD of non-replicates (same time, but different pen); All pens do not contain all features, resulting in high RSD for low intensity peaks. Whenever the intensity is high, the feature exists in the sample and is used for classifying (Figure 5c, main text) - for those features the RSD is low as it is supposed to be (grey area).

Table S1. HTX matrix configurations. The configuration yielding the highest intensity was chosen for the experiment.

Index	CHCA conc.	C°	Flowrate	Layers
1	5	75	70	8
2	10	75	70	4
3	5	80	70	8
4	10	80	70	4
5	5	75	100	8
6	10	75	100	4
7	5	80	100	8
8	10	80	100	4
